# Outcomes and Management of Dislocated Hip Hemiarthroplasty

**DOI:** 10.7759/cureus.70928

**Published:** 2024-10-06

**Authors:** Prateek A Saxena, Niyam Amanullah, Shyam Rajagopalan, Neil Ashwood

**Affiliations:** 1 Orthopedic Surgery, Queens Hospital Burton, Burton, GBR; 2 Trauma and Orthopedics, University Hospitals of Derby and Burton NHS Foundation Trust, Derby, GBR

**Keywords:** bipolar hemiarthroplasty, hip hemiarthroplasty, patient outcomes, recurrent hip dislocation, revision joint replacement

## Abstract

Purpose and aims

Hip hemiarthroplasty (HHA) is a common procedure undertaken to manage intracapsular neck of femur fractures. Dislocation of HHA is one of the most dreadful complications. There is a paucity of clinical evidence to guide decision-making for managing these patients. The aim of this study was to describe the operative management and outcomes of patients with dislocated hemiarthroplasties of the hip and outline a treatment strategy for their management.

Methods

We conducted a retrospective analysis of all the patients presenting to University Hospitals of Derby and Burton, UK with hip fractures between 2016-2022. We included all the patients who underwent a hemiarthroplasty for their fracture. We excluded patients who had malignancy and if clinical data was missing. Each operative intervention and subsequent dislocations were recorded. We recorded the following outcome measures: dislocation, surgical interventions, mortality, revision surgery, cognition status, residential status, and mobility. We also compare these outcomes with the patients who had HHA and did not sustain any dislocation.

Results

Of the 1134 patients treated with HHA during this period, 33 patients sustained dislocation. Of the 33 patients, 29 were female and 4 were male with mean ages of 87.4±7.4 and 89.25 ± 9.54,​ respectively. Following the first dislocation, 25 patients were treated with closed reduction, six patients had excision arthroplasty (EA), and two patients were treated non-operatively. About 21 patients went on to have second and third dislocations, none of these had EA and others had conversion to total hip replacement (THR). Nearly 80% of dislocations occurred within two months of the initial procedure. The mean mental test score was 7.91±2.01 (p=0.001) and was significantly higher in patients who underwent conversion to THR. The average ASA grade was significantly higher in patients who had closed reduction (2.93±0.25, p=0.001) and EA (3.28±0.46, p=0.002) compared to the patients who had no dislocation. Patients who underwent EA had significantly higher acute length of hospital stay 23.5±13.5 (p=0.02) and mortality (p=0.001) compared to other groups. We found no significant difference in dislocation rates where the initial procedure was carried out by registrars or consultants (p=0.567).

Conclusion

We concluded that the dislocation risk is higher in females and within the first two months of the index procedure. More than 80% of patients had a second dislocation following a successful closed reduction. In our cohort, 45% of patients had EA (Girdlestone procedure) and 36% had a conversion to THR. EA was associated with increased mortality rates, acute length of hospital stays, and significant change to premorbid mobility status. A multidisciplinary team (MDT) approach is necessary following the second dislocation to prevent further morbidity associated with recurrent dislocations.

## Introduction

In the UK, hip hemiarthroplasty (HHA) is one of the most commonly performed procedures for displaced intracapsular neck of femur fractures in the elderly population. In 2023, almost 77000 hip fractures were treated and 43% of these underwent HHA [1,2]. Treating elderly and frail patients is often challenging owing to their multiple co-morbidities and polypharmacy. However, careful optimization and timely undertaking of surgical procedures are recommended by the National Institute of Clinical Excellence (NICE) to help manage pain and early mobilization of these patients. HHA is a commonly performed procedure and often has a very low complication rate. Commonly seen complications following HHA are infection, periprosthetic fracture, acetabular erosion, dislocation, neurovascular injury, and leg-length inequality [3,4]. Dislocation following HHA is a rare yet major and devastating complication. The reported incidence of HHA dislocation varies between 0.8% and 15% [5-9]. Due to the low incidence rate and high mortality in the neck of femur fracture patients, there is a paucity of clinical evidence for its management in the current literature. Decision-making and management of dislocated HHA are crucial owing to increased morbidity and high costs associated with their management. Our aim was to outline the management of dislocated HHA, the morbidity and mortality associated with it, and to describe our treatment strategy.

## Materials and methods

The data for all the patients presenting to University Hospitals of Derby and Burton NHS Foundation Trust (UHDB) with neck of femur fractures was collected prospectively by the audit clerks and recorded in the database. Following their discharge, these patients were managed in the community. We conducted a retrospective review of this prospectively collected data for all the patients presenting with HHA dislocation. We included all patients who underwent primary HHA between January 2016 and October 2022 and subsequently sustained a dislocation. It is uncertain if any patients who were initially managed at UHDB and sustained a dislocation have moved out of the region or been treated in another institution. This scenario is extremely rare and unlikely as the current practice in the UK is to notify the primary surgeon and team of any arthroplasty complication. We excluded patients who had primary HHA for a pathological fracture or had any data missing. We used electronic systems such as Meditech, Lorenzo, and Cito to collect data on all the patients. We collected and analyzed their age, sex, date of injury, date of surgery, mobility status, social status, abbreviated mental test scores (AMTS), grade of operating surgeon, past medical history, complications, and time of death. In addition, we also documented the date of index dislocation and its management strategies such as non-operative, manipulation and closed reduction, revision to total hip replacement, and excision arthroplasty (EA). We documented the final treatment for each episode of dislocation and simultaneously analyzed the clinical outcomes following those treatments. The clinical outcome measures of interest in this study were the rate of dislocation, the re-dislocation rate, perioperative mortality, length of acute hospital stay, discharge destination, and mobility status.

The analysis was performed using SPSS version 23.0. The means of categorical and continuous data were analyzed using an independent student t-test and the Fisher exact test. Significance was set at p=0.05 and two tailed p values were reported. 

## Results

During this period, we treated 5521 neck of femur fractures at our institution and 1134 needed a HHA for intracapsular neck femur fractures. All the patients received cemented femoral stems and bipolar heads. All the index hemiarthroplasty was performed using the anterolateral approach to the hip with patients in the lateral position. 

**Figure 1:**
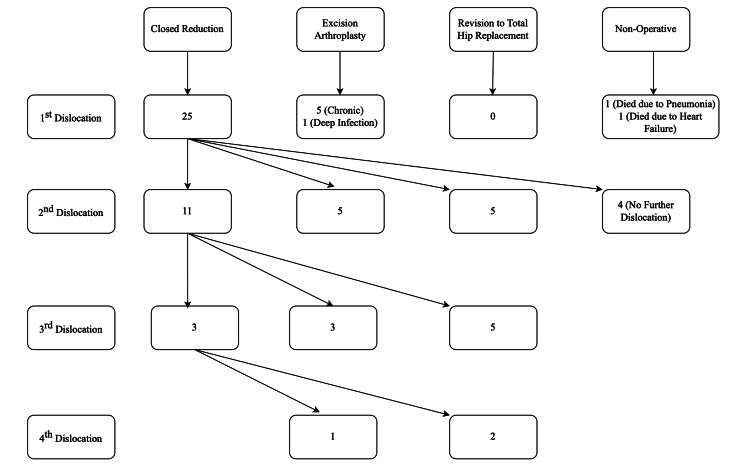
Summary of all the patients with dislocation and their management

All 33 patients (N=33), 29 females and 4 males, sustained dislocation from this cohort. Therefore, the dislocation rate in our cohort was 2.91%. The mean age for males was 89.25±9.54 yrs and for females was 87.4±7.4 yrs. The mean time to the first dislocation following index surgery was 34±24 days. At index dislocation, sic patients had EA as their definitive management due to chronic undiagnosed dislocation (N=5) and deep prosthetic joint infection (N=1). Two patients died prior to any surgical intervention due to medical co-morbidities and complications. The remaining 25 patients underwent a successful closed reduction and had clinically stable hips post-reduction. Four patients required no further treatment after their initial closed reduction. Therefore, closed reduction was a successful intervention in 16% of cases (four out of 25 cases). Subsequently, 21 of these stable hips went on to have a seconddislocation. Following a multidisciplinary team (MDT) discussion, 11 patients underwent a successful attempt at closed reduction, resulting in clinically stable hips post-reduction. Five patients underwent EA, and five were revised to a THR due to instability. All 11 patients who underwent closed reduction only following the second dislocation, sustained a third dislocation. Subsequent to the thirddislocation, five patients had revision to THR, three had EA and three underwent closed reduction with clinically stable hips post-reduction. Three patients who were managed with closed reduction after the third dislocation sustained a fourth dislocation following which two of them were revised to THR and one had EA. All the revision THR were performed by consultants and a dual mobility cup with cemented femoral stem was used. This overall management has been outlined in Figure 1.

The dislocated HHA patients were further subdivided into groups depending on their surgical management. The mean age of patients without dislocation and patients undergoing revision THR following dislocation was 84.33±8.7yrs. The mean age for patients undergoing MUA and EA was higher than THR and non-dislocated group (86±9.4 yrs and 87.45±7.45 yrs, respectively). The average age for patients undergoing EA was significantly higher compared to all the other groups of patients (p=0.03). 

The mean AMTS in non-dislocated patients was 5.9±3.4. Among the dislocated group, the mean AMTS was significantly higher in patients undergoing revision THR (7.9±2.01) compared to others (p=0.001). Although the mean AMTS score was lower in patients undergoing EA (4.64±3.81), this difference was not statistically significant (insufficient numbers). The American Society of Anesthesiologists (ASA) grade for patients who did not suffer a dislocation was 2.7±0.7. This was lower when compared with the mean ASA grade for the patients who sustained a dislocation. Within the dislocation group, the patients who were managed with revision to THR and MUA had lower ASA grades (2.9±2.5 and 2.9±0.3, respectively) compared to patients managed with EA (3.48±4.6). This difference in ASA grade was statistically significant in EA and MUA groups (p=0.002 and p=0.001). Table 1 summarizes these characteristics for each intervention group.

**Table 1:** Age, AMTS, and ASA AMTS: abbreviated mini mental test score; ASA: American Association of Anesthesiologists; THP: total hip replacement; EA: excision arthroplasty

	Non-dislocated	Closed reduction	EA	Revision to THP
Mean age (yrs)	84.33±8.7	86±9.4 (p=0.345)	87.45±7.45 (p=0.034)	84.33±8.7 (p=0.51)
Mean AMTS	5.9±3.4	6.93±3.41 (p=0.49)	4.64±3.81 (p=0.405)	7.91±2.01 (p=0.0013)
Mean ASA	2.7±0.7	2.93±0.25 (p=0.001)	3.28±0.46 (p=0.002)	2.9±0.3 (p=0.4)

**Figure 2:**
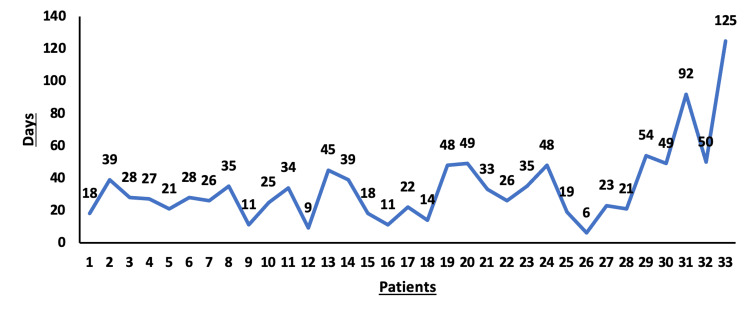
Summary of time to index dislocation for all patients in the cohort

Figure 2 summarizes the time of the first dislocation for all cases in our cohort. A total of 24 dislocations occurred early within six weeks of the index procedure, eight patients had dislocation within 90 days, and one had it late after three months of the index procedure. Despite notable differences in actual values, this was statistically insignificant (p>0.05).

**Figure 3:**
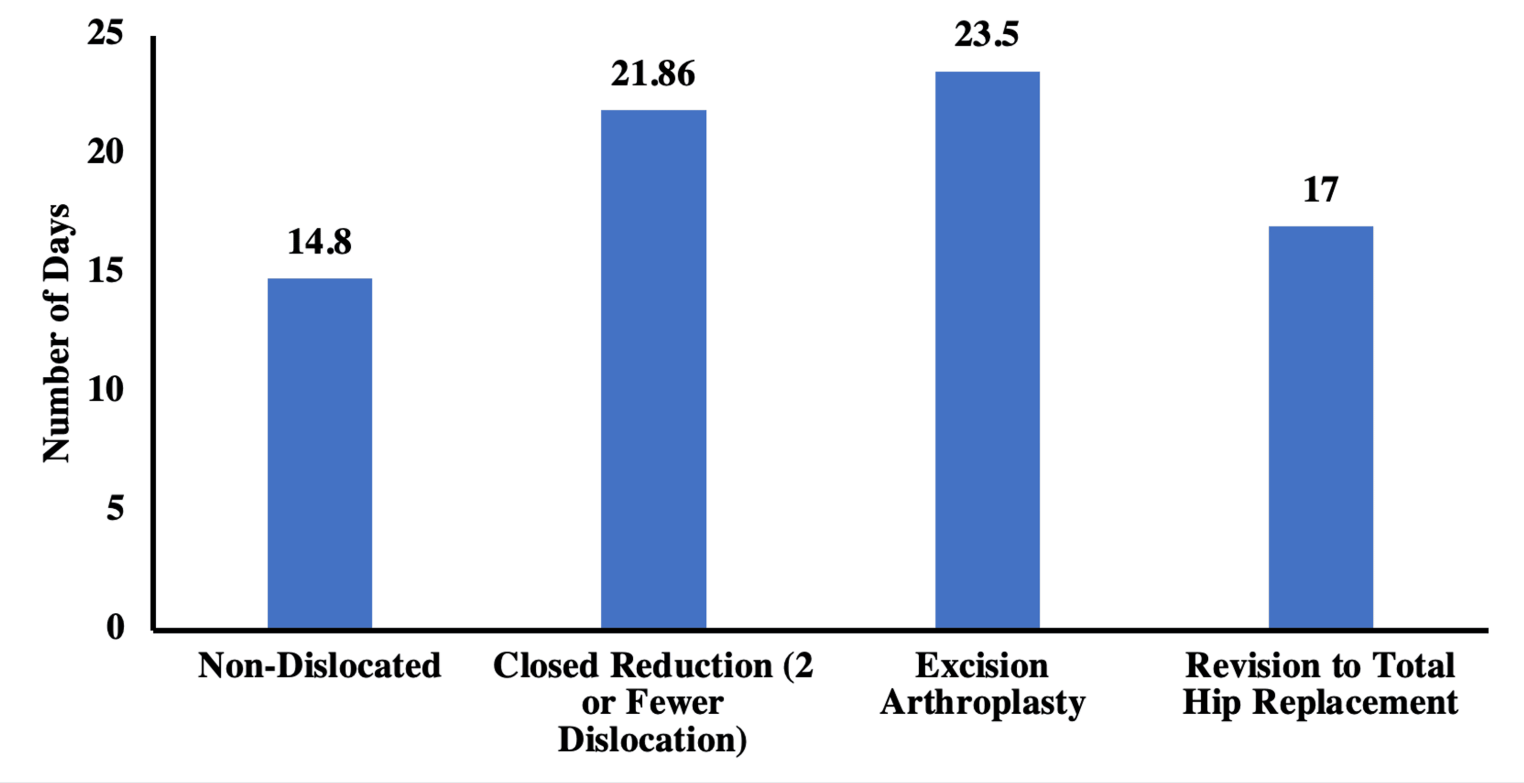
Length of hospital stay for all groups of patients with HHA dislocation HHA: hip hemiarthroplasty

The average length of stay (LOS) for patients without dislocation after HHA was 14.8 days. Generally, the average LOS was higher in patients who sustained a dislocation. The highest LOS was in patients undergoing EA (23.5 days), which was significantly higher (p = 0.02) than that of patients who had closed reduction only (21.86 days) and revision to THR (17 days), respectively. Figure 3 compares the average LOS in all the groups. 

**Figure 4:**
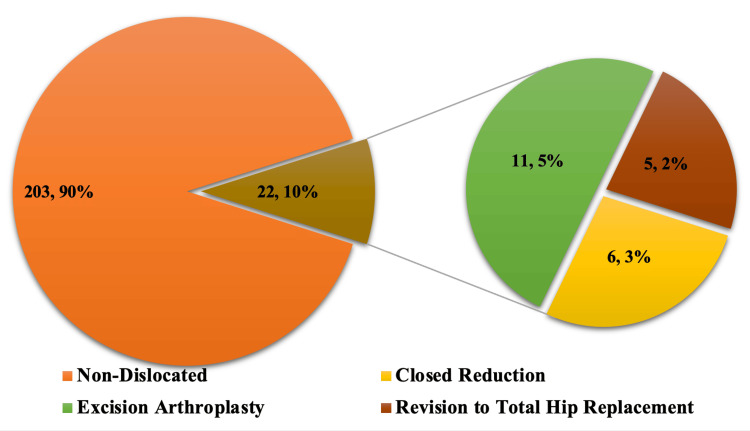
Graphic representation of overall mortality in HHA patients who sustained a dislocation HHA: hip hemiarthroplasty

The overall one-year mortality rate was 19.8% for all the patients (N=225/1134) who underwent HHA during this period. The highest mortality was in patients who underwent EA (N=11), and this was statistically significant (FE test, p=0.001). There was a lower mortality rate in patients undergoing closed reduction only (N=6) and in those who had revision to a THR (N=5) However, this was statistically not significant (p=0.118 and p=0.116, respectively). Figure 4 demonstrates the overall mortality in HHA patients and the subdivided mortality rate in patients who had dislocation.

**Figure 5:**
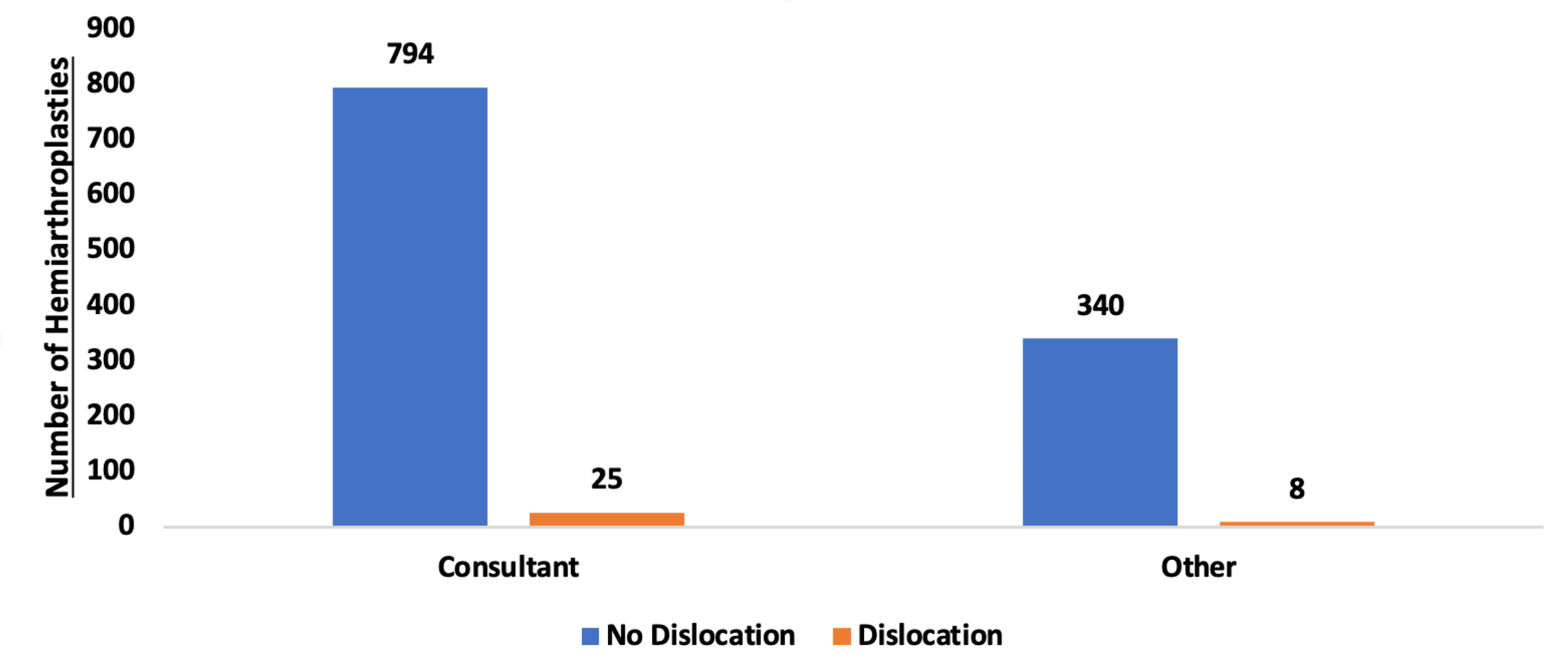
Comparison of consultant and non-consultant grade surgeons performing index hemiarthroplasty

Most of the index surgeries (N=819) were performed by the consultants and non-consultant surgeons performed only half (N=348) of the total HHA performed within our hospitals. There was no significant difference in dislocation rate while comparing these two groups of surgeons (FE test, p=0.567). Figure 5 demonstrates the difference between the two groups.

## Discussion

The dislocation rate in our cohort was 2.91%. This is keeping in the recorded range of 0.8%-6.1% in the published literature [5,7-13]. About 72% of dislocations reportedly happened within six weeks after their index surgery. In the literature, several authors have noted similar results and more than 50% of HHA dislocated within the first six weeks of index procedures [8,10,14].

ASA grade is considered a surrogate marker of fitness for surgery [15]. In our cohort, the mean ASA grade was more than two in patients who sustained an HHA dislocation. Several other studies have reported similarly higher ASA grades; however, due to limitations in their studies, they could not reach statistical significance [8,14]. However, in our cohort the mean ASA grade was higher in patients who underwent EA or MUA, concluding that the patients with high ASA grade are much more likely to receive either MUA or EA as their definitive management for recurrent dislocation of HHA. The patients with dislocated HHA who were revised to a THR had significantly lower ASA grades. So younger or more medically fit patients should be considered for a THR as a definitive procedure following recurrent dislocation.

Cognitive impairment has been noted to have a negative impact on postoperative rehabilitation and recovery, and it also impedes the patient’s ability to consent to the procedure. Thus, as per national guidelines, all the patients are screened at the time of admission for any cognitive impairment using the AMTS tool [16]. Patients with lower AMTS scores due to any form of cognitive impairment are at high risk of dislocation, as evident from our case series [17]. 

As per current literature, more than 50% of patients sustain recurrent HHA dislocation after the first successful primary reduction [18]. For a recurrent THR dislocation, the current guidelines are to consider revision surgery [10,19]. This same approach is not usually employed for HHA in current UK practice, despite the high incidence of recurrent dislocation following the first successful reduction. Due to an overall low dislocation rate, high mortality rate, poor soft tissue tension, and low demand among the neck of femur fracture patients, there is insufficient evidence to formulate such strategies for this group of patients. Closed reduction alone is a good and successful initial intervention, but it has a high re-dislocation rate and is rarely successful as the definitive procedure, as evident from the literature and our case series [8,20]. However, it is evident from our case series that closed reduction alone is only successful in 16% of cases. Thus, an MDT to plan a definitive procedure following the second dislocation is vital due to a very high re-dislocation rate following index dislocation. 

Nearly 48% of patients in our cohort had a revision of HHA to a THR and demonstrated good mobility and survivorship. The current literature supports good short- and mid-term outcomes with excellent survivorship following the conversion of HHA to a THR for recurrent dislocations [20,21]. Patients with an ASA grade of less than 3, age less than 85 years, and an AMTS score of 7 should be considered for conversion to THR. Revision to THR has been shown to reduce the LOS in the hospital and help reduce further morbidity associated with dislocated HHA. Through an MDT approach, several other factors could be considered prior to this decision-making. In 2023, NICE recommended that these surgical interventions should be considered as a part of the palliative care approach, as they help minimize pain and other symptoms and help to establish individual rehabilitation goals.

EA has been associated with high mortality rates and very poor outcomes [22]. Previous studies and our case series outline these poor outcomes, especially the high mortality rate and prolonged LOS in hospital among this group of patients [8]. This option should be reserved for very elderly and medically frail or unfit patients.

There are a number of limitations to our study. We conducted a retrospective analysis of a small cohort of patients, and this could result in type 2 errors affecting the outcomes. The study reviewed patients over six years, and we may not have been able to include all of them, especially if they had moved out of the region or were treated in a different institution. Due to the low incidence, changing hospital systems, changing implants, and heterogenicity of treatments for each case, radiological analysis was difficult for each case to conclude any anatomical factors such as femoral anteversion, femoral neck cuts, and head size.

**Figure 6:**
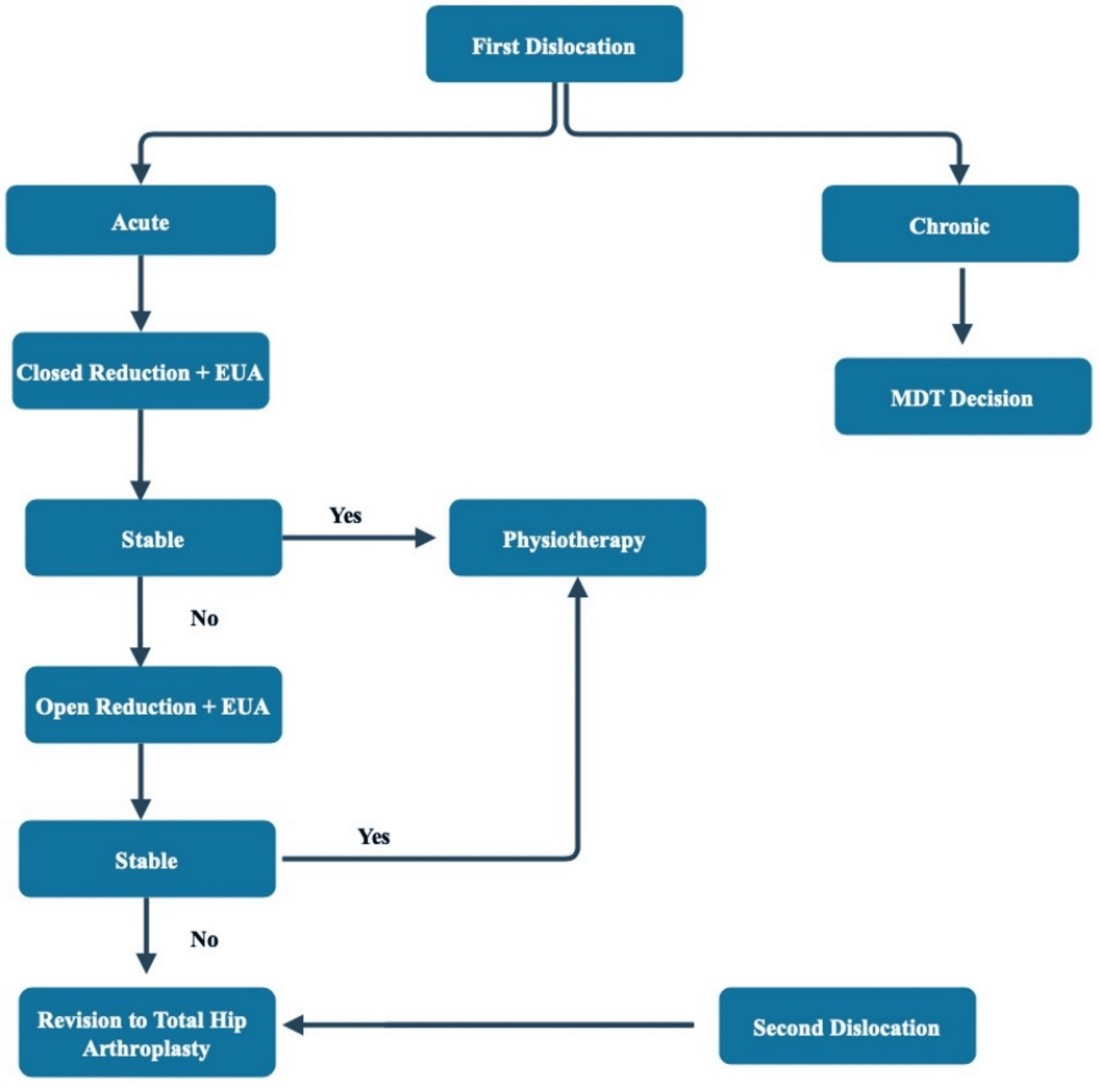
Algorithm for management of HHA dislocation EUA: examination under anesthesia; MDT: multidisciplinary team; HHA: hip hemiarthroplasty

Based on the current findings from our study, we have outlined an algorithm in Figure 6 for managing HHA dislocation in elderly patients and currently using it in our clinical practice.

## Conclusions

The risk of dislocation is higher in females. It is highest within six weeks of the index procedure, and this decreases with time. Re-dislocation rate is high (80%) after the successful first closed reduction and nearly one-third of re-dislocation after the second closed reduction attempt, thus revision to THR should be considered after the first dislocation. EA should be avoided as the first treatment and should be only considered as a last resort in recurrent dislocation following HHA, due to long length of hospital stay and high mortality rates. Non-consultant grade surgeons performing the index procedures do not influence the dislocation rate.

There is no standardized decision-making considering the choice of treatment following HHA dislocation, and we recommend an individualized MDT approach for each recurrent HHA dislocation case.
